# Human Cytomegalovirus Manipulation of Latently Infected Cells

**DOI:** 10.3390/v5112803

**Published:** 2013-11-21

**Authors:** John H. Sinclair, Matthew B. Reeves

**Affiliations:** 1 Department of Medicine, University of Cambridge, Addenbrooke’s Hospital, Hills Road, Cambridge, CB2 0QQ, UK; E-Mail: js152@cam.ac.uk; 2 Institute of Immunity and Transplantation, Division of Infection and Immunity, University College London, Royal Free Campus, Rowland Hill Street, London, NW3 2PF, UK

**Keywords:** cytomegalovirus, latency, immune evasion, apoptosis, gene expression, cellular signalling

## Abstract

Primary infection with human cytomegalovirus (HCMV) results in the establishment of a lifelong infection of the host which is aided by the ability of HCMV to undergo a latent infection. One site of HCMV latency *in vivo* is in haematopoietic progenitor cells, resident in the bone marrow, with genome carriage and reactivation being restricted to the cells of the myeloid lineage. Until recently, HCMV latency has been considered to be relatively quiescent with the virus being maintained essentially as a “silent partner” until conditions are met that trigger reactivation. However, advances in techniques to study global changes in gene expression have begun to show that HCMV latency is a highly active process which involves expression of specific latency-associated viral gene products which orchestrate major changes in the latently infected cell. These changes are argued to help maintain latent infection and to modulate the cellular environment to the benefit of latent virus. In this review, we will discuss these new findings and how they impact not only on our understanding of the biology of HCMV latency but also how they could provide tantalising glimpses into mechanisms that could become targets for the clearance of latent HCMV.

## 1. Introduction

Human Cytomegalovirus (HCMV) remains a major cause of disease in a number of patient populations who have compromised immune systems, as well as providing an increasing threat to critically ill immuno-competent patients [1,2,3,4]. These pathologies associated with opportunistic HCMV infections can be, in part, associated with a key characteristic of the virus: the ability to establish lifelong latent infection of the human host and, crucially, reactivate [2,5]. A wealth of studies from a number of laboratories using naturally latently infected cells has led to an informed consensus that the cells of the myeloid lineage represent at least one important site of HCMV latency, persistence, and reactivation (reviewed in [6]). Thus, at a cellular level, there is a clear and intimate link between myeloid differentiation and natural HCMV reactivation [7,8,9,10,11,12,13,14]. Furthermore, the use of experimental infection of non-permissive primary cells and cell lines *in vitro* are generating snapshots of the complex regulation of HCMV gene expression at a molecular level [15,16,17,18,19,20,21,22,23,24,25]. However, these studies have focussed predominantly on the regulation of major immediate early (MIE) gene expression because the critical switch to a reactivating phenotype is dependent on the triggering of MIE gene expression from quiescence.

In many cases, the species specificity of HCMV has driven these analyses to be performed in experimental cell culture models and, ultimately, on tissue derived from healthy HCMV seropositive individuals which has then been analysed *ex vivo*. As a result, the mechanisms that control HCMV latency and persistence *in vivo*, at an organism level, have relied on the extrapolation of studies performed *in vitro* or using animal model surrogates such as murine CMV [26]; guinea pig CMV [27] and, more recently, non-human primate CMV strains [28]. Consequently, the inability to perform analogous studies in humans has likely contributed to the perception that HCMV latency is essentially a relatively quiescent infection. However, as techniques for studying HCMV at a molecular level have become increasingly powerful, it is now emerging that latent HCMV infection profoundly modulates the latently infected cell and the surrounding cellular environment. These effects act in concert to maintain latent carriage and this depends on, at least in part, the expression of a subset of virally encoded gene products. 

In this short review, we will examine our current knowledge of HCMV latency with particular emphasis on recent data which suggest that HCMV imparts a distinctive signature on latently infected cells. These latency-associated changes underpin the successful persistence of this virus *in vivo* and, importantly, could direct novel therapeutic strategies to target latency and reactivation of this important human pathogen. 

## 2. Background—HCMV Latency and Reactivation

Following primary infection, HCMV establishes a latent infection of the CD34+ haematopoietic cell population in the bone marrow [29,30]. The prevailing view is that, ultimately, the major immediate early promoter (MIEP) is profoundly suppressed in these cells [6] and that this is achieved through cellular transcriptional repressors directing histone-modifying enzymes to impart repressive post-translational modifications of MIEP-associated histones [6]. During latency, the chromatin structure of the MIEP bears all the hallmarks of transcriptional repression: tri-methylation of histone H3 (lysine 9 and 27) and recruitment of heterochromatin protein-1 (HP-1) coupled with a concomitant absence of histone acetylation on histone H4 [11,16,17,25]. Consequently, HCMV MIE gene expression, and lytic gene expression in general, is profoundly repressed in CD34+ progenitor cells. This chromatin phenotype is maintained in the monocyte cells derived from these progenitors [11,31] and it is only upon cellular differentiation that robust IE gene expression is observed [7,8,11,12,32]. The detection of IE gene expression in dendritic cells (DCs) is consistent with the histone modifications present at the MIEP in these terminally differentiated myeloid cells [11,31]. For instance, HP-1 is no longer associated with the MIEP—likely due to extensive de-methylation of histones at lysine residue 9 (methylation at this residue being important for HP-1 binding to chromatin [33]) and, in these cells, the MIEP is associated with predominantly acetylated histones. Thus, the presence of repressive or activatory chromatin marks around the MIEP correlates with the expression of viral major IE RNA and the latency/reactivation phenotype of the virus [11,31]. Importantly, and consistent with molecular analyses, infectious HCMV progeny cannot be recovered from myeloid progenitor cells *i.e.*, CD34+ cells or granulocyte–macrophage progenitors (GMPs) unless they are co-cultured under conditions that promote cellular differentiation or activation [9,11,34]. Analogous models of histone-mediated regulation of viral lytic gene expression also underpin studies of herpes simplex virus and Epstein–Barr virus and thus represent a common unifying theme in the biology of herpesvirus latency and reactivation [35,36]. 

The molecular model of HCMV latency in the myeloid lineage, derived from analyses of natural latency, has been reviewed extensively elsewhere [6,37,38] and has helped provide an initial understanding of the underlying mechanism for the differentiation-dependent reactivation of HCMV. It is worth noting, however, that other studies using experimental infection models of latency and reactivation have essentially recapitulated the key observations made with natural models of latent infection and this gives confidence that wider studies involving experimentally latent models will have *in vivo* relevance.

### 2.1. The Transcriptional Landscape of Latent HCMV

HCMV encodes anywhere between 170 and 751 ORFs all of which are believed to be expressed at some stage during lytic infection [39,40]. Furthermore, the virus also encodes a number of microRNAs (miRNAs) which, during lytic infection, have been shown to target and regulate both cell and viral gene expression [41,42,43]. In contrast, the transcriptional landscape in latency is less clear. The earliest studies identified a number of transcripts arising from the MIE region of HCMV but no function was assigned to them [44,45]. Furthermore, deletion of the putative ORFs encoded by these latency-associated transcripts appeared to have little effect on HCMV latency *in vitro* [46]. As such, it was speculated that HCMV could exist in latency in a relatively quiescent state and that the normal transit and differentiation of latently infected CD34+ cells into the periphery was sufficient to trigger HCMV reactivation. Indeed, transcriptional quiescence during latency would provide the ideal mechanism for evasion of the robust immune responses known to be present in HCMV seropositive individuals [47]. However, a number of aspects of the known biology of HCMV are at odds with the view that HCMV is maintained in a totally quiescent state. For instance, if virus is carried long-term in the myeloid lineage, how is the latent genome maintained in cells which will, at least at some stage of their lifespan, proliferate? Although no latent origin of replication has been definitively identified for HCMV, it has been suggested that a mutation in the MIE region had a carriage defect during latency in GMPs [48] and more recent work has suggested UL84 may act to maintain viral sequences [25]. Furthermore, an overt characteristic of HCMV latency is the carriage of the viral genome in the cells of the myeloid lineage and, particularly, the monocyte lineage [49,50,51] but not lymphocyte or polymorphonuclear cells [50] despite the fact that latent infection is seeded in a pluripotent progenitor cell type [29,30]. Potentially, this could be explained in alternative ways: the virus actively promotes myelopoiesis of infected CD34+ cells or, HCMV may preferentially promote the survival of myeloid committed progenitors or, finally, HCMV cannot combat anti-viral mechanisms in cells committed to the lymphoid lineage. Arguably, all these scenarios suggest an active process involving viral latency-associated functions during latent infection.

A number of studies over the last 10 years or so have applied increasingly sensitive techniques to determine whether viral gene expression occurs during latent infection. Two independent microarray analyses identified a number of transcripts expressed during experimental latency [34,52] and, importantly, some have been subsequently confirmed during natural latency; including UL138, UL81-82ast (LUNA), as well as a splice variant of UL111A, which encodes a viral interleukin 10 (vIL-10) termed LAcmvIL-10 [24,53,54,55]. These, and subsequent studies, have also shown that the initial infection of undifferentiated myeloid cells with HCMV to establish experimental latency results in a burst of temporally dysregulated viral transcription from a number of gene loci, including MIE gene expression, at very early times *post* infection [25,32,34]. However, it remains unclear what this means in the context of latent infection. It is tempting to speculate that this gene expression is important for preparing the cell for latency—akin to that proposed for the establishment of EBV latency [56]. However, there is no evidence, as yet, that cells which initially express lytic antigens go on to establish long-term latency. It is possible that the extremely high MOIs used to establish latent infections *in vitro* results in a sub-population of lytically or abortively infected cells which are, ultimately, unviable and die, leaving the true latent population. 

Regardless, what is generally accepted is that HCMV has a very distinct transcriptional profile during latent infection, quite different from lytic infection. The expression of a number of viral genes has now been described during latency and these are summarised in Table 1. For the remainder of this review, we will focus on emerging stories regarding the manipulation of latently infected cells by HCMV and how, in some instances, viral gene products may contribute to this.

**Table 1:** Gene products and functions during latency and lytic infection.

Gene Product	Latent Function	Lytic Function	References
**CLTs**	Unknown	Regulation of anti-viral 2’5’ OAS expression (ORF94)	[44,45,46,57]
**UL138**	Regulation of TNFRI (up) and MRP1 (down), repression of the MIEP(?)	Regulation of TNFRI (up) and MRP1 (down), virus maturation (133-138 locus)	[53,58,59,60,61]
**UL81-82ast**	Promotes UL138 gene expression.	Unknown	[24,55,62]
**LAvIL-10**	Down-regulation of MHC class II expression, immune evasion	Unknown—cmvIL-10 expressed during lytic infection	[54,63]
**Lnc4.9**	Binds Polycomb repressor complex 2, Silencing of the MIEP	Unknown	[25]
UL84	**Genome maintenance**	**DNA replication, UTPase activity, transcriptional regulation**	**[25,64,65,66,67]**
**US28**	Unknown	GPCR, induces cell signalling and cell migration, agonist of the MIEP	[68,69,70,71,72,73,74]
**UL144**	Unknown	TNF superfamily member, hijacks NF-kB signalling, immune evasion?	[75,76,77,78]

## 3. Mechanisms Targeted during HCMV Latency

### 3.1. Viral Evasion of Cell Death

Pro-death signals in response to infection represent a very significant obstacle for many pathogens. Consequently, key players in the cellular apoptotic response become important targets for the virus—and HCMV is no exception. HCMV encodes an impressive armoury of anti-apoptotic functions that it expresses throughout lytic infection and which all contribute to efficient virus infection [79,80,81,82,83,84]. However, there is no evidence that any of these already-described anti-apoptotic viral genes associated with lytic infection are also expressed during latency. Clearly, if the virus was to be carried truly silently during latency then, arguably, there would be little requirement for any increased protection from cell death. However, it is becoming increasingly evident that HCMV does actively modulate multiple functions of the latently infected cell and that these, in effect, stress the cell to the point that viral functions are needed to protect the latently infected cell from such stress-induced pro-death signals. 

In the context of infection, be it latent or lytic, the initiation of cell death can arise at the earliest point of infection: at entry [85]. Pathogen recognition receptors (PRRs) can detect pathogen-associated molecular patterns (PAMPs), triggering cell death—and this is an important part of an intrinsic immune response [85]. Clearly, during lytic infection, the rapid expression of virally encoded anti-apoptotic proteins could quickly provide protection against such extrinsic death response signals [80]. However, during HCMV infection of cells destined to become latently infected with the associated suppression of the lytic transcription programme, it appears that virus binding, in itself, activates cell survival signals [86,87]. This occurs in both CD34+ cells and CD14+ cells, albeit with the employment of different signalling pathways in the two cell types as well as cell-specific differences in the duration of the survival response. Nevertheless, the up-regulation of an important cellular anti-apoptotic protein, MCL-1 [88], appeared to be important for protection in both cell types [86,87]. Thus, although the exact mechanisms of protection varied in these different cell types, the outcome was the same. 

**Figure 1:**
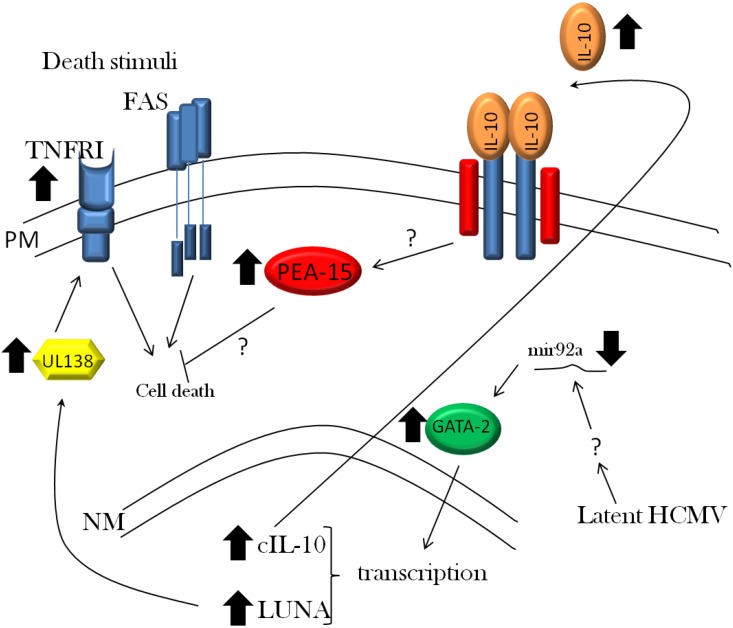
Protection of latently infected cells from cell death. CD34+ cells latently infected with HCMV down-regulate the expression of mir92a. A key target of miRNA is the GATA-2 transcription factor which, consequently, is up-regulated. This promotes increased transcription of cellular (IL-10) and viral (LUNA) genes. LUNA expression promotes UL138 gene expression—a gene product shown to up-regulate cell surface levels of TNFRI, a potentially pro-apoptotic signalling factor. However, HCMV also up-regulates a number of anti-apoptotic factors including PEA-15. This occurs, in part, via the expression of IL-10 and potentially could provide a mechanism to protect cells from extrinsic cell death signalling.

The transitory nature of the ERK-MAPK dependent survival signal observed in CD34+ cells [86] argues that is likely to be important for overcoming the initial death signals triggered by cellular recognition of virus shortly after binding and/or entry. Consequently, it could be argued that long-term anti-death signals may not be required by a virus which is truly silenced in latency. However, recent work suggests that long-term anti-death signals may be important during latent infection with HCMV (Figure 1). For instance, experimental latent infection of granulocyte–macrophage progenitors has been shown to result in long-term up-regulation of PEA-15 RNA [89]. As PEA-15 is an anti-apoptotic factor that blocks both TNFR1 and Fas-L triggered apoptosis [90], clearly its up-regulation could be part of a protective response mediated by latent infection. Consistent with this, latently infected CD34+ cells are protected from FAS-L induced cell death [91]. Furthermore, given that it has been shown that the UL138 gene product up-regulates TNFR1 expression during lytic [59,60] and latent infection [61], potentially sensitising latently infected cells to TNFR1 mediated apoptosis, the concomitant up-regulation of PEA-15 would be a sensible pro-survival strategy. Other preliminary data from the Sinclair laboratory has also shown that a number of other cellular proteins with potent anti-apoptotic function are up-regulated in latently infected CD34+ cells (J.S. unpublished data) and this includes the PEA-15 protein, further supporting a model by which induction of PEA-15 during latent infection, at least in part, protects latently infected cells from pro-death signals. Furthermore, it is likely that these effects are driven by secreted products in the latency-associated secretome [92], since inhibition of latency-induced cellular IL-10 was sufficient to block this survival effect [91].

### 3.2. Viral Evasion of the Immune Response during Latent Infection

**Figure 2:**
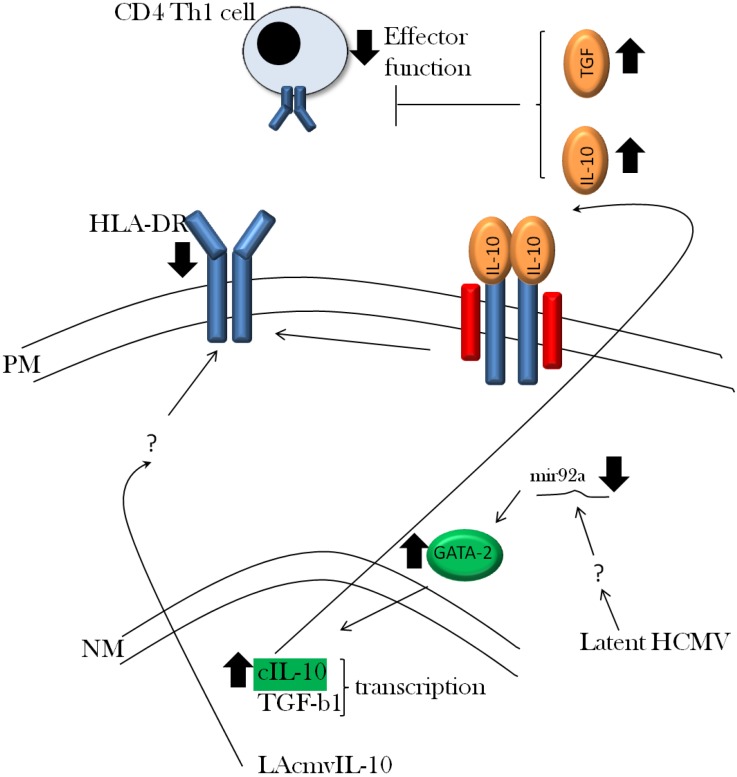
Evasion of the immune response to HCMV. The up-regulation of IL-10 in latently infected CD34+ cells is concomitant with TGF-b up-regulation via an unknown mechanism. However, the expression of two potent immune-suppressive cytokines inhibits the effector functions of CD4 Th1 cells recruited to a latently infected cell. Furthermore, both cellular IL-10 and viral IL-10 (LAcmvIL-10) act in concert to promote the down-regulation of HLA-DR MHC class II molecules on the surface of latently infected cells. Although the mechanism used by LAcmvIl-10 is not yet understood but is known not to occur via binding to the cellular IL-10 receptor.

HCMV infection is known to generate a robust T cell response *in vivo* with between 0.5 and 10% of all cytotoxic T lympohcytes (CTLs) recognising HCMV antigens [47]. The CTL response to HCMV is dominated by two abundant viral antigens—pp65 and IE72 [47], although CTLs which recognise most, if not all, lytic antigens have been detected [93]. Pertinent to this review is that significant T cell responses against antigens also expressed during latency are present in healthy HCMV carriers [94,95] and thus, in theory, a latently infected cell should be visible to these T cells. However, recent work suggests that latent infection results in a number of mechanisms which act in concert to disrupt these T cell responses, thereby preventing clearance of latently infected cells by the adaptive arm of the host immune response (Figure 2).

A recent analysis of experimentally latently infected CD34+ cells detected a unique cell secretome signature associated with latency [92]. Intriguingly, this secretome was observed to promote the migration of Th1 CD4+ T cells to the latently infected cell. However, the anti-viral effector functions of these recruited cytotoxic T cells was countered by the concomitant latency-associated expression of two key cellular cytokines, transforming growth factor—beta (TGF-β) and interleukin-10 (cIL-10). Both TGF-β and cIL-10 have profound immune-modulatory capacity [96] and, consistent with this, blocked the CD4+ T effector functions [92]. Although the exact mechanisms that resulted in up-regulated expression of TGF-β and cIL-10 during latency are unclear, elevated cIL-10 production was observed to be, at least partly, dependent on the up-regulation of the cellular GATA-2 transcription factor resulting from a concomitant down-regulation of the cellular microRNA mir92a [91]. Furthermore, other recent work has illustrated that a proportion of the CD4+ T cell response directed against latent antigens consists of T regulatory (Treg) cells [95]. This study in healthy donors showed that, whilst cytotoxic CD4+ T cell responses against latent antigens were detectable, they were dominated by IL-10 expressing Treg cells. Consequently, the recruitment of Treg cells to a latently infected cell (74) would augment the effects of the immune-suppressive secretome around the latently infected cells dampening down CTL effector cell function [92]. Given the extremely low frequency of latently infected cells in a healthy seropositive individual [97], it is likely that the microenvironment around a latently infected cell would have little overall impact on the normal immune homeostasis of the bone marrow but may be locally sufficient to ensure latently infected cells evade elimination by the immune system.

Induction of cIL-10 by latent virus clearly appears to be of real import for latent carriage and this view is, perhaps, reinforced by the fact that HCMV also encodes an IL-10 homolog, known as cmvIL-10, which is expressed solely during lytic infection, as well as an alternatively spliced form (LAcmvIL-10) expressed during both latent infection and lytic infection [98,99]. Interestingly, lytic infection-associated cmvIL-10 has retained many of the immune-suppressive functions associated with its cellular counterpart [100,101,102] and, indeed, signals via the human IL-10 receptor [98,103]. Consistent with a role for cmvIL-10-mediated immune evasion are studies in rhesus CMV that have demonstrated a role in viral dissemination [104]—presumably via a temporary dampening of the immune response. It is tempting to speculate that failure to evade the immune response during a primary infection could profoundly impact on the set point of latency but, unfortunately, this has not been possible to analyse.

As stated above, the cmvIL-10 gene encodes a number of biological properties that could impinge on HCMV latency and reactivation. Multiple studies have shown that cmvIL-10 promotes MHC class I and II down-regulation [102], prevents DC maturation and function [105,106] and promotes the polarisation of macrophages to an M2c phenotype [107]—which is considered to be a relatively inactive macrophage phenotype compared with the classic inflammatory M1 phenotype. As such, all these functions would be consistent with a role in immune evasion. However, the alternatively spliced LAcmvIL-10, though also detected during lytic infection, is the isoform expressed during latent infection [54] but does not exhibit many of the properties of cmvIL-10 [63]—presumably, in part, due to its inability to bind the cIL-10 receptor [63]. Crucially, however, both latently infected GMPs and monocytes have been shown to exhibit a dramatic decrease in cell surface expression of MHC class II [32,108]—a function associated with LAcmvIL-10 [63]. Importantly the deletion of the UL111A locus from the virus (and thus LAcmvIL-10) has illustrated that latently cells become sensitive to CD4+ recognition and killing [109] as well as impacting on the normal differentiation of myeloid progenitor cells to a DC phenotype [110]. Thus despite a loss of many of the functions associated with cmvIL-10, the LAcmvIL-10 isoform has retained biological properties that could contribute to successful persistence during latency *in vivo*.

### 3.3. Viral Regulation of Immediate Early Gene Expression

As already discussed, the regulation of HCMV MIE gene expression during latency involves the action of higher order chromatin structure. As such, it has been hypothesised that the assembly and modification of histones at the MIEP is an intrinsic response dictated by the cellular environment. Indeed, at low MOIs during lytic infection, there appears to be pre-immediate early gene expression event where the MIEP is associated with methylated histones [111]. This may well represent an anti-viral response to foreign DNA that is mediated by ND10 bodies and their components and is overcome by the action of incoming viral pp71 tegument protein and, subsequently, newly expressed IE72 which has been reviewed extensively elsewhere [112,113,114]. In contrast to lytic infection, the intrinsic repression of the MIEP is not overcome in non-productive myeloid cells. One study has proposed that unknown mechanisms that exclude pp71 from the nucleus in CD34+ cells contributes to this [23], although the high levels of transcriptional repressors present in these cells is also likely to be important; consistent with this, the transfected MIEP is intrinsically less active in undifferentiated myeloid cells [115]. Indeed, a number of transcriptional repressors of the MIEP have been identified (such as YY1 and ERF) and these are believed to recruit histone methyltransferases [116,117] to the MIEP in undifferentiated myeloid cells and this is important for generating the signature repressive chromatin phenotype associated with the MIEP of latent HCMV [11]. 

**Figure 3:**
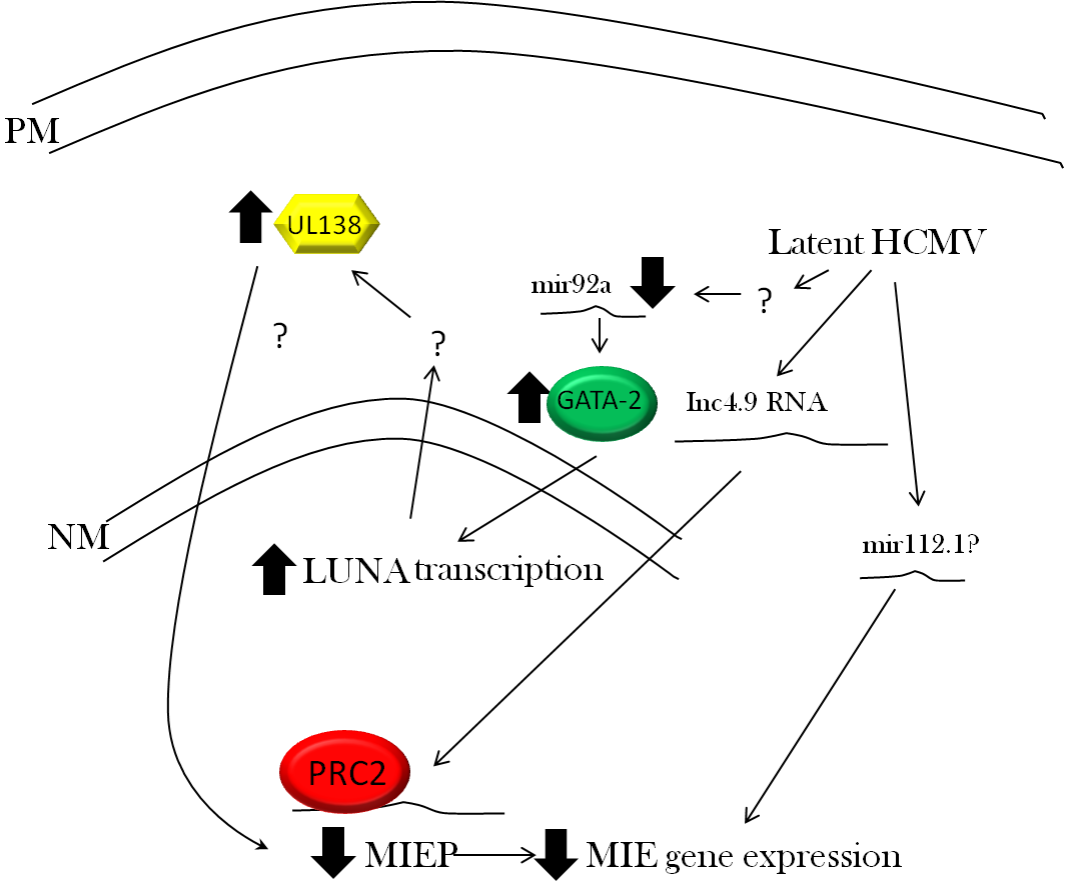
HCMV mediated repression of IE gene expression. Latent infection of CD34+ cells is characterised by a repression of the major immediate early promoter (MIEP). Classically, the MIEP has been shown to be repressed by multiple cellular transcriptional repressors known to interact with components of the histone modifying enzyme families. However, these events may be augmented by the activity of further viral mechanisms. The expression of LUNA during latency has been shown to be important for UL138 expression—a protein postulated to repress the MIEP. Furthermore, the expression of the long non-coding 4.9kb RNA (lnc4.9 RNA) during latency has been suggested to promote the recruitment of polycomb repressor complex 2 (PRC2) to the MIEP via direct binding of the RNA. Recruitment of PRC2 would promote a chromatin structure inhibitory for MIE transcription. Finally, the expression of a viral miRNA, mir112.1, has been hypothesised to be important for silencing translation from MIE transcript UL123 during latency.

However, more recent work suggests that HCMV gene products themselves may be actively helping to manage MIE regulation during HCMV latency. Although the prevailing view of the MIEP during latency in CD34+ cells is a promoter predominantly associated with repressive chromatin marks (*i.e.*, histone methylation and HP-1 binding), chromatin and its post-translational modification is highly dynamic. Studies analysing the chromatin state of well-characterised silenced cellular genes, in e.g., stem cells, suggest that all cellular promoters bear at least some hallmarks of transcription [118]. Histone methylation at lysine 4 (a marker of a recently transcribed promoter) has been identified at “silent promoters” and, consistent with this, small RNA fragments were identified which would correspond to aborted transcription events [118]. Thus the notion of “chromatin breathing,” even at repressed promoters, is not uncommon. Given the potent activity of the MIEP, there is a strong argument that the MIEP is unlikely to be completely transcriptionally repressed, even in the most undifferentiated myeloid cell, and that this will call for additional mechanisms to eliminate any residual low level, uncontrolled MIE expression (Figure 3).

During lytic infection, HCMV expresses a virally encoded microRNA (mirUL-112-1) that specifically target IE72 encoding UL123 transcripts [119,120] and inhibits IE72 translation [120]. Deletion of mirUL-112-1 has no overt phenotype in infected fibroblasts, likely due to the substantial levels of IE72 transcript accompanying lytic infection [120]. However, it has been postulated that low levels of IE72 RNA may be targeted efficiently and, hence, miRUL112-1 may have more of a role during latency [119]—where untimely IE72 expression could be problematic but where the less abundant levels of IE72 RNA could be more effectively controlled by a miRNA-mediated mechanisms. In effect, the microRNA acts as a safety net to ensure that the functional impact of any sporadic activity of the MIEP, and any resultant IE transcripts, are minimised during latent infection. 

A more recent study [25] that used a deep sequencing approach to re-visit latent gene expression in experimental as well as naturally latent tissue samples, identified the expression of a number of viral transcripts including a 4.9 kb long non-coding RNA (lnc4.9). Interestingly, this transcript was observed to associate with the polycomb repressor complex 2 (PRC2)—with direct analogy to the KSHV PAN RNA species that also bind this complex [121]. Indeed, the PRC2 complex has also been shown to regulate HSV latency, although this is thought to occur independently of direct binding to the LAT RNA [122]. Furthermore, the binding of components of PRC2 and also the lnc4.9 RNA was observed in experimentally latently infected cells [25]. The net result of such interactions would be to augment the silencing of the viral MIEP linked with histone tri-methylation at lysine 27 on histone H3. The overall contribution of the lnc4.9 RNA to HCMV latency remains to be determined; however, analysis of whether virus mutants that fail to express lnc4.9 are defective in their establishment and maintenance of latency could help determine whether this interaction is as an essential component of the mechanisms required to maintain HCMV latency. 

Finally, other recent work has also suggested that the MIEP may repressed by a virally encoded factor during latency [23]. Treatment with histone deacetylase inhibitors (HDACi) is insufficient to promote the reactivation of IE gene expression in CD34+ cells latently infected with clinical strains of HCMV [23]. Conversely, however, CD34+ cells latently infected with laboratory isolates are responsive to HDACi. Consequently, this has suggested that a viral factor present in clinical isolates is involved in chromatin-mediated suppression of MIEP activity during experimental latency [23]. The likely candidate is UL138, which is expressed only in clinical isolates [39] and is known to be expressed during latent infection [34,53]. However, published studies suggest that the importance of UL138 for latency is not due to any direct effect on MIEP activity as a transcriptional repressor [58], thereby remaining in line with the predominant localisation of UL138 protein to the Golgi apparatus during lytic infection and transfection [58]. One caveat to this, though, is that the localisation of the UL138 protein has not been extensively analysed during latent infection and hence, at this stage, a role for UL138 in the repression of the viral MIEP during latency awaits further analyses. 

### 3.4. Viral Regulation of Latent Gene Expression

In addition to the regulation of MIE gene expression, there is emerging evidence that HCMV also expresses functions to ensure efficient latent gene expression during latent infection (Figure 3). This, in itself, argues that latent viral gene products are likely to have important functions during latency and, importantly, that these could act as potential therapeutic targets for latent infection.

The modulation of the cellular miRNAome during latent infection could provide potent fine-tuning mechanisms to optimise both viral and cellular gene expression [91]. As discussed earlier, the down-regulation of cellular hsa-miR-92a by HCMV is important for the increased cIL-10 production that results in downstream effects on viability and immune modulation [91,92]. However, the down-regulation of hsa-miR-92a also results in an increase in the levels of the GATA-2 transcription factor [91]. The GATA family of proteins are considered key regulators of haematopoiesis and myeloid cell production [123,124] and, thus, the targeting of this transcription factor in the knowledge that HCMV persists in the myeloid lineage appears more than coincidental. However, a more direct effect of GATA-2 regulation is observed on latent gene expression. A number of promoters of latently expressed genes contain consensus sequences for GATA-2 binding sites [77,91,125,126] and two of these, LUNA and UL144, have been directly demonstrated to be GATA-2 responsive [77,126]. Recent work has shown that the down-regulation of the hsa-mir92a observed in HCMV infected CD34+ cells results in increased GATA-2 levels during latency, subsequently leading to increased levels of GATA-2-dependent latent gene expression [91]. 

Although we are far from completely elucidating the function of viral gene products during latency, observations, to date, strongly argue that latent infection with HCMV results in a latency-associated transcription profile of viral gene expression, resulting in an orchestrated change in the cell to support latent carriage. The regulation of latent viral gene expression, as well as the role of latent viral functions and their effects on cellular gene expression, are clearly inextricably linked. For instance, recent work from the St Jeor laboratory has shown that the expression of LUNA during latent infection is also important for latency-associated UL138 gene expression [62]. Consequently, a pathway of interactions appears to occur during latency which is exemplified by latency, thus resulting in the targeting of cellular hsa-miR92a; this, in turn, up-regulates cellular GATA-2 expression [91], leading to a downstream impact on latency-associated LUNA gene expression [77,91] and ultimately ensuring the expression of UL138 [62], which has been proposed to be a key determinant of latency [23,53].

Clearly, this simplified example of a linear pathway of viral and cellular interactions is likely to give way to far more complex networks of host–virus interactions as we begin to understand the multi-functional role of viral proteins, non-coding RNAs, and miRNAs during HCMV latency and their impact on the latent cell. 

## 4. Concluding Remarks

The advances in molecular techniques for performing large-scale analyses at the cell level are allowing ever more detailed analyses of aspects of HCMV biology which, previously, were all but impossible due the limitations of sensitivity and the availability of tractable primary cell models. These approaches have already begun to illustrate the complexity of HCMV latency and to provide an intriguing view of the concerted efforts HCMV employs to maintain the latent state. 

It is evident that the reductionist approach of these types of studies, as well as the difficulty in further examining *in vitro* findings *in vivo*, warrants necessary caution to prevent overinterpretation. Accordingly, a key development in the future of HCMV studies of latency and reactivation will be the tractability and applicability of the humanised mouse model to studies of HCMV [127]. Caveats with this system also remain; although the humanised mouse can be used to assess HCMV reactivation in the myeloid lineage *in vivo*, this is still occurring in the background of mouse tissue that does not support extensive HCMV replication [128]. Consequently, such analyses are, arguably, restricted to the very initial events of HCMV reactivation occurring within a specific niche of human cells. Furthermore, the extent to which the human haematopoietic system develops from engrafted human CD34+ cells in the mouse (for instance, murine and human cytokines do not crosstalk unequivocally) is unclear and, more generally, the extent to which mouse models of disease truly reflect the human condition is an area of ongoing debate [129,130]. Nevertheless, the humanised mouse model could provide the potential to examine a number of predictions regarding HCMV latency derived from *in vitro* studies, as well as certain aspects of the development of the immune response to latent HCMV. 

These cautionary notes aside, the identification of viral gene functions expressed during experimental latency (many of which, importantly, can be validated in naturally latent cells *ex vivo*) is beginning to provide a tantalising glimpse into the once-perceived “black box” of latency. As we begin to understand the functions of these latency-associated gene products, and assess their precise role in HCMV latency and reactivation, they are also likely to become potential targets for therapeutics. These approaches could range from the targeting of factors important for HCMV reactivation (our own unpublished work suggests that the LUNA gene product may encode a function that could be a future therapeutic target) or for the direct targeting of latently infected cells using chemotherapeutic or immunotherapeutic means [61]. 

Anti-viral strategies for HCMV have, to date, relied on targeting of replicating virus during lytic infection. However, understanding the complex interplay between the virus and the host during latency will give important insights into how to explore potential therapeutic options that target latent virus in what was previously considered to be in an “untargetable state.”
